# Computational, experimental details, and biological raw data accompanying the publication: “The synthesis and characterization of a nanomagnetite with potent antibacterial activity and low mammalian toxicity”

**DOI:** 10.1016/j.dib.2018.08.097

**Published:** 2018-09-11

**Authors:** Seyedeh Maryamdokht Taimoory, Abbas Rahdar, Mousa Aliahmad, Fardin Sadeghfar, Mohammad Reza Hajinezhad, Mohammad Jahantigh, Parisa Shahbazi, John F. Trant

**Affiliations:** a Department of Chemistry and Biochemistry, University of Windsor, 401 Sunset Avenue, Windsor, ON, Canada N9B 3P4; b Department of Physics, University of Zabol, P. O. Box. 35856-98613, Zabol, Iran; c Department of Physics, University of Sistan & Baluchestan, Zahedan, Iran; d Basic Science Department, Faculty of Veterinary Medicine, University of Zabol, Zabol, Iran; e Department of Clinical Sciences, School of Veterinary Medicine, University of Zabol, Zabol, Iran

## Abstract

This data file includes experimental details on how to make uncoated iron oxide nanoparticles using a green electrochemical method. It provides the raw data on the antibacterial activity of one of these formulations, and the full computational data and methodology used to generate that data, of several different magnetite clusters of specific spin multiplicities for 4, 5, 7 and 9 iron atom magnetite clusters. This data will assist other researchers wishing to replicate or expand on these results for the investigation and use of nanomagnetite for antibacterial applications.

**Specifications table**

**Table:** 

Subject area	*Chemistry and materials science*
More specific subject area	*Iron oxide nanoparticles, characterization and biological activity*
Type of data	*Experimental details, tables of biological results,.txt files for use as computational input files*.
How data was acquired	*Computational data was acquired using Gaussian09, biological data was accomplished using a variety of optical techniques described in the experimental sections below.*
Data format	*Raw biological data, calculated computational data*
Experimental factors	*All samples were prepared according to the protocols included.*
Experimental features	*All experiments were conducted according to the protocols included.*
Data source location	*Not relevant*
Data accessibility	*Data included in this article*
Related research article	*Accompanying a submitted article to Molecular Liquids, Submitted Jan 05, 2018.*

**Value of the data**

• The biological data shows potent activity. The data is provided here for other researchers to compare with their own as magnetite is further investigated as a potential antibacterial material.
• The computational results are the most detailed study of magnetite in the literature to the best of our knowledge. It is our hope that other researchers can use these as a starting point for even better refining the computational models in the future.
• This is a scalable, green, inexpensive synthesis of nanomagnetite with low dispersity. The synthesis described here will hopefully be helpful to others looking to replicate or make similar systems.

## Data

The data includes the experimental protocols required to make the nanomagnetite, the experimental details for both the computational and biological studies, the biological data used for our analyses, and full co-ordinates for all the computational models referenced in the accompanying article: “The Synthesis and Characterization of a Nanomagnetite with Potent Antibacterial Activity and Low Mammalian Toxicity [1].” We hope that this data release will assist other researchers investigating similar systems and similar applications.

## 1 Experimental design, materials, and methods from the publication

### 1.1 General experimental information

Iron (II) sulfate heptahydrate (FeSO_4_·7H_2_O, Sigma-Aldrich) and sodium hydroxide (Sigma-Aldrich) were analytical grade and used as received without further purification. The DU145 human adenocarcinoma cell line was purchased from Pasteur Institute (Iran). X-ray powder diffraction was carried out on a D8 Advance X’Pert X-Ray diffractometer (Bruker); Fourier-transform infrared spectroscopy was done on a JASCO 640 plus machine (4000–400 cm^−1^) at room temperature using KBr pellets; vibrating magnetometry was analyzed using a Kavir Precise Magnetic instrument (MDKFT, Iran); field emission scanning electron microscopy was carried out using a Mira 3-XMU instrument capable of 700,000× magnification.

### 1.2 Nanoparticle synthesis

The magnetite nanoparticles were prepared using a surfactant-free electrochemical approach as previously reported in a closed distilled water system, without inert gas atmosphere [2]. Additional details are provided in the SI.

### 1.3 Computational methodology

The computational calculations for our proposed iron oxide clusters (**Fe**_**4**_, **Fe**_**5**_, **Fe**_**7**_, and **Fe**_**9**_) were performed using the Gaussian 09 suite of programs [3]. Additional details are provided in the SI.

### 1.4 Ethics review

The following biological research was all approved by the ethics board at the University of Zabol [2], [3], [4], [5], [6], [7], [8]. The animal studies were conducted following the guidelines of the Animal Ethics Committee of the Faculty of Veterinary Medicine, University of Zabol, Zabol, Iran [2], [3], [4], [5], [6], [7], [8]. All animal treatment was carried out in a humane fashion in compliance with both institutional and EU Directive 2010/63/EU requirements.

### 1.5 Antibacterial assay

The determination of the minimum inhibitory concentration (MIC) was carried out in 96-well plates using serial dilutions of a stock of sonicated 3000 MP Ultrasonic Homogenizer nanoparticles (those prepared as above at pH = 13) according to the standard protocol [4]. *Escherichia coli* and *Staphylococcus aureus* were prepared from human diagnostic laboratories and identified using standard bacteriological methods [5]. Additional details are provided in the SI.

### 1.6 Animal studies

All weights are dry weights. Adult male Wistar rats (190–210 g) obtained from the Laboratory Animal Center University of Zabol were used in this study. Rats were randomly divided into four groups (*n* = 10). The control group was given 1 ml of distilled water using the oral gavage technique once a day for 14 days. The second group was treated with 1 ml of nanomagnetite solution (10 ppm/ml/day) once a day, for 14 days. The third group received 1 ml of nanomagnetite solution (100 ppm/ml/day) once a day, for 14 days. The fourth group received 1 ml of nanomagnetite solution (1000 ppm/ml/day) once a day, for 14 days. At the end of the feeding schedule (14 days) the animals were sacrificed by cervical dislocation and the blood samples were collected from the heart and centrifuged (3000 rpm for 5 min) to recover the serum. The serum was then immediately frozen at −80 °C until needed for the assays detailed in the supporting material and described below.
