# Supplementary material for: Computational, experimental details, and biological raw data accompanying the publication: “The synthesis and characterization of a nanomagnetite with potent antibacterial activity and low mammalian toxicity”
Source: Data Brief. 2018 Sep 11;21:2518–21. doi: 10.1016/j.dib.2018.08.097 (PMC6288310; doi:10.1016/j.dib.2018.08.097)
Supplement: Supplementary file 1 — Supplementary material [file mmc1.pdf]

Conflict of Interest and Authorship Conformation Form

Please check the following as appropriate:

- ✓ All authors have participated in (a) conception and design, or analysis and interpretation of the data; (b) drafting the article or revising it critically for important intellectual content; and (c) approval of the final version.

- ✓ This manuscript has not been submitted to, nor is under review at, another journal or other publishing venue.

- ✓ The authors have no affiliation with any organization with a direct or indirect financial interest in the subject matter discussed in the manuscript

- ✓ The following authors have affiliations with organizations with direct or indirect financial interest in the subject matter discussed in the manuscript:

Author's name	Affiliation

The authors declare no conflicts of interest with regards to this work.
