# Supplementary material for: Computational, experimental details, and biological raw data accompanying the publication: “The synthesis and characterization of a nanomagnetite with potent antibacterial activity and low mammalian toxicity”
Source: Data Brief. 2018 Sep 11;21:2518–21. doi: 10.1016/j.dib.2018.08.097 (PMC6288310; doi:10.1016/j.dib.2018.08.097)
Supplement: Supplementary file 2 — Supplementary material. [file mmc2.docx]

**Supplementary Information for Data Article: Computational, experimental details, and biological raw data accompanying the publication “****The synthesis and characterization of a magnetite nanoparticle with potent antibacterial activity and low mammalian toxicity”**

Seyedeh Maryamdokht Taimoory^a^, Abbas Rahdar^b,*^, Mousa Aliahmad^c^_,_ Fardin Sadeghfar^c^, Mohammad Reza Hajinezhad ^e^, Mohammad Jahantigh^f^, Parisa Shahbazi^f^, John F. Trant^a,*^

*^a^ Department of Chemistry and Biochemistry, University of Windsor, Windsor, ON N9B 3P4, Canada; ^b^ Department of Physics, University of Zabol, Zabol, Iran; ^c^Department of Physics, University of Sistan & Baluchestan, Zahedan, Iran; ^e^Assistant Professor of physiology, Basic Science Department, Faculty of Veterinary Medicine, University of Zabol, Zabol, Iran; ^f^ Department of Clinical Sciences, School of Veterinary Medicine, University of Zabol, Zabol, Iran*

1. **Experimental Details**

***1.1 Nanoparticle Synthesis:*** The distance between the anode (stainless steel) and the cathode (stainless steel) was maintained at 6 cm for all syntheses. The reaction solution was prepared by dissolving iron sulfate heptahydrate (4g, 14.4 mmol) in 200 mL of doubly distilled water to provide a 72 mM solution. Two steel plates (13x23x0.5 mm) were then electroplated with the solution at 130.43 mA·cm^-2^ for 3 hours. This created the initial layer of iron oxide.[[1](#_ENREF_1)] The anode and cathode were then replaced, and a solution of sodium hydroxide (1 M) was then added dropwise to raise the pH to the desired level. The electrochemical reaction was then allowed to continue for 3 hours at 4.31 mA·cm^-2^ before the current was removed. The resulting nanoparticles deposited on the iron-coated steel plates were then collected, and were extensively washed with double distilled water through iterative rounds of suspension, vortexing, centrifugation, and decanting. The material was then dried for 2 hours at 60 ºC, and stored until analyzed.

***1.2 Computational Methodology***: Our computational models were generated with GaussView and optimized using the DFT hybrid B3LYP (RHF and UHF methods), functional (Becke 3-term correlation functional; Lee, Yang, and Parr exchange function) with the 6-31G** and/or 6-311++G** basis set at 298.15 K (25 °C), or using the faster semiempirical quantum-chemical method, РМ6 at different spin multiplicities.[[2](#_ENREF_2)] All of the optimized clusters were verified by frequency computations as minima (zero imaginary frequencies). From these values we were able to graph the simulated IR spectrum. The crystallographic data of the magnetite iron oxide (Fe_3_O_4_, ICSD 43001) was obtained from the report by Ma and co-workers.[[3](#_ENREF_3)] The cubic magnetite unit cell of a cluster containing 56 atoms (Fe_24_O_32_) was found to be a sufficient structure to model our synthetic iron oxide nanoparticles (Figure 1). From this structure, as reported in our pervious work,^10^ a 3D crystal structure visualization and a theoretical XRD spectrum were generated and simulated using VESTA (Visualization for Electronic and STructural Analysis).[[4](#_ENREF_4)]

***1.3 Antibacterial Assay:*** The determination of the minimum inhibitory concentration (MIC) was carried out in 96-well plates using serial dilutions of a stock of sonicated (3000MP Ultrasonic Homogenizer) nanoparticles (prepared as above at pH =13) according to the standard protocol.[[5](#_ENREF_5)] *Escherichia coli* and *Staphylococcus aureus* were prepared from human diagnostic laboratories and identified using standard bacteriological methods.[[6](#_ENREF_6)] Additional details are provided in the SI. Healthy colonies of E. coli and S. aureus were selected from the agar plates and transferred to a glass tube containing 5 mL of saline solution (0.9%), and the solution was vortexed and was visually determined, by inspection, to be equivalent turbidity to a 0.5 MacFarland standard. This solution was used as the stock solution of the organisms. Positive controls (ampicillin and chloramphenicol, Sigma-Aldrich) were used in the study. Negative control (broth media and colonies) showed no inhibition of bacterial growth, demonstrating the health of the colonies. Following the serial dilutions of the nanoparticles (stock solution = 90 mg/mL) and the antibiotics (ampicillin stock solution = 10 mg/mL and chloramphenicol stock solution = 20 mg/mL), the organism was added to the each well and the plates were incubated at 37 ºC for 24 hours. The plates were removed and the wells were visually inspected for the MIC of nanoparticles and the antibiotics. All media and solutions were obtained from Padtan Teb Company (Iran).

***1.4.1 Animal in vivo Studies***: Animals were kept at 22–24°C under 12-hour light/dark cycles. The rats were housed in polycarbonate cages with free access to sterile tap water and a standard pellet diet (Javaneh Khorasan Company, Mashhad, Iran).

***1.4.2 Animal in vivo Assay Analysis:*** Serum catalase activity was measured using a commercial enzymatic kit (ZellBio GmbH, Ulm, Germany). In this assay, a unit of catalase activity is defined as the volume of the serum sample required to catalyze the decomposition of one micromole of H_2_O_2_, to water and O_2_, in one minute.[[7](#_ENREF_7)] Lipid peroxidation was measured according to the method previously described by Okhawa.[[8](#_ENREF_8)] This method is based on the reaction between malondialdehyde and 2-thiobarbituric acid (TBA). The absorbance of the red colored oxidized TBA product was measured at 532 nm (UNICO UV/VIS- 2100 Spectrophotometer). Malondialdehyde concentration was then calculated based on the standard curve in ηmol/ml.[[9](#_ENREF_9)] Statistical analyses were performed using SPSS software (version 20.0). Multiple comparisons were performed by ANOVA and Dunnett tests. The critical difference (CD) was set at P<0.05.

Alanine aminotransferase (ALT) and aspartate aminotransferase (AST) levels were measured using a Pars Azmoon commercial reagent kit (Pars Azmoon Co., Tehran, IRI) on a Selectra Pro M autoanalyzer (Vital Scientific, SpanNeren, Netherlands) according to the manufacturer’s recommended protocol.

Serum creatinine and blood urea nitrogen (BUN) levels were measured using similar commercial reagent kits (Pars Azmoon Co., Tehran, IRI) on a Selectra Pro M autoanalyzer (Vital Scientific, SpanNeren, Netherlands) according to the manufacturer’s recommended protocol.

1. **Computational Details and Co-Ordinates:**

***2.1 Parameters for the Fe7(H) and Fe9(H) clusters.***

**
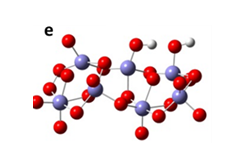
**
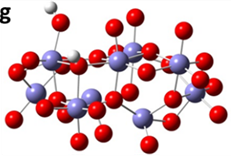


**Fe7(H) Fe9(H)**

**Fe-Fe =** 2.71 Å **Fe-Fe =** 2.78 Å

**E**(PM6) **=** -861.1591146 -1216.678207 kcal/mol

**Figure S1.** Calculated geometries, energies, and Fe-Fe bond lengths of e) **Fe7(H)** and g) **Fe9(H)**

***2.2 DFT Calculated Coordinates and Thermochemical Data***

**2.2.1 Fe4 iron oxide cluster ( *M* = 15)**

-----------------------------------------------------------------

# opt=calcfc freq=noraman ub3lyp/6-31++g(d) geom=connectivity temperature=298

Zero-point correction= 0.229271 (Hartree/Particle)

Thermal correction to Energy= 0.260185

Thermal correction to Enthalpy= 0.261129

Thermal correction to Gibbs Free Energy= 0.167810

Sum of electronic and zero-point Energies= -6267.734036

Sum of electronic and thermal Energies= -6267.703121

Sum of electronic and thermal Enthalpies= -6267.702178

Sum of electronic and thermal Free Energies= -6267.795496

0 15

Fe 3.04213400 -0.58380000 -0.35838300

Fe -2.45426100 0.30490900 -0.94871100

O 1.28669600 -0.26898400 0.41478900

O 0.47629300 -2.82508100 -0.03702600

O 0.61458200 1.84215600 -1.07293200

O 2.09294700 2.53172800 0.92239600

O -1.72850100 -1.46553500 -1.05538900

O -3.76002500 -2.50089100 1.21268800

O -1.06534100 0.35273900 0.49451200

O -0.76839000 3.05653400 0.79609300

O -2.88300800 2.07550900 -0.74382400

O 3.05070500 -2.39177500 0.04316400

O 3.48981400 -0.66960900 -2.08314500

O 3.81986000 0.80430800 0.06786500

O -3.94324600 -0.20821800 -0.17776000

O -1.55632500 0.69329500 -2.49275300

Fe 0.44532700 1.40462800 0.64467200

Fe -0.28213800 -1.33689900 0.41674700

O 0.43643200 1.30175900 2.45391000

O -1.14665600 -1.94139100 1.92003500

H 2.21583600 2.45102300 1.88748200

H -1.38522700 -1.67847100 -1.94167000

H -2.81637100 -2.47902000 1.50822000

H -0.30539400 3.73894000 0.27691800

H -3.72559500 2.10450400 -0.25378000

H 3.58225000 -2.93816100 -0.55904200

H 3.98225900 0.11835400 -2.37091600

H 2.85583400 2.00510400 0.50989500

H -3.91245100 -1.09432800 0.30332000

H -1.85386500 1.54948100 -2.84934400

H -1.31328200 -1.16641500 2.48911500

H 0.06923900 1.30239300 -1.68882100

H -0.32020800 1.79565700 2.81616400

H -1.56559800 2.80439700 0.24916300

H -3.85047500 -3.30185600 0.67410400

H 1.48873600 -2.81377300 0.03574400

1 12 1.0 13 1.0 14 1.0 3 1.0

2 7 1.0 11 1.0 15 1.0 16 1.0 9 1.0

3 17 1.0 18 1.0

4 18 1.0 36 1.0

5 17 1.0 32 1.0

6 21 1.0 28 1.0 17 1.0

7 22 1.0

8 23 1.0 35 1.0

9 17 1.0 18 1.0

10 24 1.0 34 1.0 17 1.0

11 25 1.0

12 26 1.0

13 27 1.0

14

15 29 1.0

16 30 1.0

17 19 1.0

18 20 1.0

19 33 1.0

20 31 1.0

21

22

23

24

25

26

27

28

29

30

31

32

33

34

35

36

- - 1. **Fe4 iron oxide cluster ( *M* = 17)**

-----------------------------------------------------------------

# opt=calcfc freq=noraman b3lyp/6-311++g(d,p) geom=connectivity temperature=298

----------------------------------------------------------------

Zero-point correction= 0.225260 (Hartree/Particle)

Thermal correction to Energy= 0.258171

Thermal correction to Enthalpy= 0.259115

Thermal correction to Gibbs Free Energy= 0.161531

Sum of electronic and zero-point Energies= -6268.489320

Sum of electronic and thermal Energies= -6268.456408

Sum of electronic and thermal Enthalpies= -6268.455465

Sum of electronic and thermal Free Energies= -6268.553049

0 17

Fe 2.90716000 -0.37421300 -0.61731200

Fe -2.53204600 0.02126800 -0.75441400

O 1.16442500 -0.09029800 0.11632200

O 0.54064100 -2.87019400 0.20562900

O 0.18712100 2.10993400 -1.10526100

O 1.98534600 2.81747700 0.73050300

O -1.67743700 -1.71856500 -0.87285900

O -2.08782200 -2.69651200 1.65484100

O -1.29923400 0.21977700 0.59229600

O -1.26334700 2.94517100 1.03156700

O -3.32202500 1.55405300 -0.15003100

O 3.01847800 -2.13042000 0.00019000

O 3.57015300 -0.69921100 -2.25488900

O 3.55215100 1.13091700 -0.37389200

O -4.07281100 -0.52910800 -1.50305900

O -1.50110700 0.59905800 -2.30068200

Fe 0.33044300 1.53005400 0.53637300

Fe -0.34638000 -1.43105500 0.64922300

O 0.50854700 1.33077000 2.36851500

O 0.10164300 -1.37784600 2.38700300

H 2.24298000 2.73765700 1.65932100

H -1.21757600 -1.77240400 -1.72337300

H -1.65459900 -2.73097500 2.51936300

H -1.12602000 3.71050400 0.45758400

H -4.22875200 1.64282700 -0.47130500

H 3.60289100 -2.68173700 -0.53326000

H 4.05837200 0.05704900 -2.60237800

H 2.69321200 2.30946900 0.20971600

H -4.02132100 -1.44955100 -1.79257800

H -2.02177300 0.93027300 -3.04079100

H 0.31730000 -0.44086900 2.60374300

H -0.39139500 1.53029600 -1.70684400

H -0.21334700 1.79828900 2.81103500

H -2.09973300 2.52252400 0.70995300

H -2.05709200 -3.58979800 1.29257200

H 1.54044100 -2.73098200 0.23708300

1 3 1.0 12 1.0 13 1.0 14 1.0

2 9 1.0 11 1.0 15 1.0

3 17 1.0

4 18 1.0 36 1.0

5 17 1.0 32 1.0

6 21 1.0 28 1.0

7 22 1.0

8 23 1.0 35 1.0

9 18 1.0

10 24 1.0 34 1.0

11 25 1.0

12 26 1.0

13 27 1.0

14

15 29 1.0

16 30 1.0

17 19 1.0

18 20 1.0

19 33 1.0

20 31 1.0

21

22

23

24

25

26

27

28

29

30

31

32

33

34

35

36

- - 1. **Fe5 iron oxide cluster ( *M* = 19)**

--------------------------------------------------------------------

# opt=calcfc freq=noraman ub3lyp/6-31g(d) geom=connectivity temperature=298

---------------------------------------------------------------------

Zero-point correction= 0.309220 (Hartree/Particle)

Thermal correction to Energy= 0.349999

Thermal correction to Enthalpy= 0.350943

Thermal correction to Gibbs Free Energy= 0.237143

Sum of electronic and zero-point Energies= -7835.810824

Sum of electronic and thermal Energies= -7835.770044

Sum of electronic and thermal Enthalpies= -7835.769100

Sum of electronic and thermal Free Energies= -7835.882900

0 19

Fe 0.02717500 0.16579400 0.30245300

Fe 2.49217100 -1.40749200 0.32065000

Fe 2.29789900 1.86366900 -0.42122700

Fe -2.80536900 -1.25213200 -0.98014300

O 0.55500700 1.50697400 -1.11504800

O 0.79057100 -1.26723500 -0.68930200

O 1.89601300 0.44875700 0.66071600

O -0.76952000 1.69750000 1.08012200

O -1.62370300 0.00696800 -0.45612900

O -0.19618400 -0.78903000 1.73711600

O 4.38602500 -0.58884800 1.17325700

O 3.20429600 -3.52259900 -0.01954800

O 3.64206700 -1.23267500 -1.40788300

O 2.99263400 1.09885100 -2.08567700

O -1.49468300 -2.41740900 -1.56111600

O 2.18871400 3.61769400 -0.75905600

O 3.85147700 2.07298400 0.47896500

O -3.03645000 -2.27922000 0.44936200

O -3.89373200 -1.57539900 -2.34151000

H 4.14556700 2.99977900 0.47333700

H -2.38829300 -3.00294300 0.35917100

H -3.66390500 -2.36731800 -2.85548800

H 1.31124400 3.81570000 -1.13153400

H 3.65154700 1.66822000 -2.51250300

H 4.28475300 -0.79696400 2.11782400

H 4.31831800 0.39759200 1.10256300

H -1.11930300 -0.79826700 2.18636300

H -1.37850600 -2.34377200 -2.52318600

H -0.70946500 1.72862700 2.04942900

H 0.71311000 1.06621400 -1.97149800

H 4.58163400 -1.26809500 -1.16439900

H 3.46288900 -0.29496600 -1.81820500

H 2.84245100 -4.00724200 -0.77810700

H 2.75886500 -3.84318500 0.79034300

Fe -2.74269100 1.47531400 0.38471300

O -4.03684600 0.06130800 -0.33994300

H -4.64602700 0.32380600 -1.05090600

O -3.31268800 1.53827100 2.16979300

O -2.52258100 -0.94910700 2.86488900

H -2.93349300 -0.04727300 2.79019400

H -4.24340300 1.76172700 2.32271200

H -2.95557000 -1.47998300 2.16189900

O 2.15169500 -2.26396200 1.90998300

H 1.30839800 -1.91123200 2.26679000

O -3.20896700 2.89994900 -0.64553400

H -2.89879100 3.79264900 -0.43538100

H 0.05400700 -1.87725000 -0.97467200

1 6 1.0 7 1.0 8 1.0 9 1.0 10 1.0

2 43 1.0

3 5 1.0 7 1.0 16 1.0 17 1.0

4 9 1.0 15 1.0 18 1.0 19 1.0 36 1.0

5 30 1.0

6 47 1.0

7

8 29 1.0

9

10

11 25 1.0 26 1.0

12 33 1.0 34 1.0

13 31 1.0

14 24 1.0

15 28 1.0

16 23 1.0

17 20 1.0

18 21 1.0

19 22 1.0

20

21

22

23

24

25

26

27

28

29

30

31

32

33

34

35 38 1.0 45 1.0

36 37 1.0

37

38 41 1.0

39 40 1.0 42 1.0

40

41

42

43 44 1.0

44

45 46 1.0

46

47

- 1. ***PM6 Calculated Coordinates and Thermochemical Data***
     1. **Fe7 iron oxide cluster ( *M* = 1)**

---------------------------------------------------------------

# opt=calcfc freq=noraman upm6 geom=connectivity temperature=298

----------------------------------------------------------------

Zero-point correction= 0.087950 (Hartree/Particle)

Thermal correction to Energy= 0.119313

Thermal correction to Enthalpy= 0.120256

Thermal correction to Gibbs Free Energy= 0.031392

Sum of electronic and zero-point Energies= -1.242927

Sum of electronic and thermal Energies= -1.211565

Sum of electronic and thermal Enthalpies= -1.210621

Sum of electronic and thermal Free Energies= -1.299485

-4 1

Fe(PDBName=FE,ResName=UNL,ResNum=1) 3.48791200 -1.11758300 0.49839900

Fe(PDBName=FE,ResName=UNL,ResNum=1) -1.63289800 -0.89491600 0.89880000

Fe(PDBName=FE,ResName=UNL,ResNum=1) 0.36593300 1.31483500 0.06502000

Fe(PDBName=FE,ResName=UNL,ResNum=1) -3.75165100 -0.87370700 -0.49571700

Fe(PDBName=FE,ResName=UNL,ResNum=1) -2.41157400 1.58033200 0.28404400

Fe(PDBName=FE,ResName=UNL,ResNum=1) 1.24530200 -1.16240700 -0.67212700

Fe(PDBName=FE,ResName=UNL,ResNum=1) 3.09310500 1.25602000 -0.38759400

O(PDBName=O,ResName=UNL,ResNum=1) 2.21951600 -2.38204600 0.21177200

O(PDBName=O,ResName=UNL,ResNum=1) 2.95806100 -0.47866400 -1.22900600

O(PDBName=O,ResName=UNL,ResNum=1) 1.88646700 0.06271800 0.66823800

O(PDBName=O,ResName=UNL,ResNum=1) -3.72952200 -1.66304900 -1.94648200

O(PDBName=O,ResName=UNL,ResNum=1) -0.07708400 -1.59726100 0.36989300

O(PDBName=O,ResName=UNL,ResNum=1) 1.72236900 1.84378400 -1.19414900

O(PDBName=O,ResName=UNL,ResNum=1) -5.18924300 -1.33302200 0.17231800

O(PDBName=O,ResName=UNL,ResNum=1) 1.21704500 -1.98380400 -2.09667100

O(PDBName=O,ResName=UNL,ResNum=1) 0.35696400 0.16427600 -1.34759900

O(PDBName=O,ResName=UNL,ResNum=1) -2.75950500 -2.19186900 0.28632400

O(PDBName=O,ResName=UNL,ResNum=1) 4.69726700 -1.98838800 -0.19708700

O(PDBName=O,ResName=UNL,ResNum=1) 4.41849800 0.36924700 0.55540100

O(PDBName=O,ResName=UNL,ResNum=1) 3.48133200 -1.31300700 2.12327200

O(PDBName=O,ResName=UNL,ResNum=1) -1.06484200 0.76978900 1.29894400

O(PDBName=O,ResName=UNL,ResNum=1) -1.78407700 -1.36331900 2.45032300

O(PDBName=O,ResName=UNL,ResNum=1) -2.02679500 -0.16686100 -0.77532900

O(PDBName=O,ResName=UNL,ResNum=1) 0.84796700 2.08439200 1.38287200

O(PDBName=O,ResName=UNL,ResNum=1) -1.10687800 1.91819100 -0.86120800

O(PDBName=O,ResName=UNL,ResNum=1) -3.33132400 0.00796400 1.06656100

O(PDBName=O,ResName=UNL,ResNum=1) -4.36539600 0.67358000 -1.24172700

O(PDBName=O,ResName=UNL,ResNum=1) -3.24663200 2.24162300 1.53207400

O(PDBName=O,ResName=UNL,ResNum=1) -3.75553700 1.89699000 -1.00352700

O(PDBName=O,ResName=UNL,ResNum=1) 4.13832500 1.67838700 -1.60692300

O(PDBName=O,ResName=UNL,ResNum=1) 3.20560400 2.41698600 0.76153500

1 8 1.0 9 1.0 18 1.0 19 1.0 20 1.0

2 12 1.0 17 1.0 21 1.0 22 1.0 23 1.0

3 13 1.0 16 1.0 24 1.0 25 1.0

4 11 1.0 14 1.0 17 1.0 23 1.0 26 1.0 27 1.0

5 21 1.0 25 1.0 28 1.0 29 1.0

6 8 1.0 9 1.0 10 1.0 12 1.0 15 1.0 16 1.0

7 13 1.0 19 1.0 30 1.0 31 1.0

8

9

10

11

12

13

14

15

16

17

18

19

20

21

22

23

24

25

26

27 29 1.0

28

29

30

31

- - 1. **Fe7 iron oxide cluster ( *M* = 5)**

--------------------------------------------------------------

# opt=calcfc freq=noraman upm6 geom=connectivity temperature=298

--------------------------------------------------------------

Zero-point correction= 0.082262 (Hartree/Particle)

Thermal correction to Energy= 0.115805

Thermal correction to Enthalpy= 0.116749

Thermal correction to Gibbs Free Energy= 0.021503

Sum of electronic and zero-point Energies= -1.293431

Sum of electronic and thermal Energies= -1.259888

Sum of electronic and thermal Enthalpies= -1.258944

Sum of electronic and thermal Free Energies= -1.354190

-4 5

Fe(PDBName=FE,ResName=UNL,ResNum=1) 3.54159200 -1.08406400 0.50829000

Fe(PDBName=FE,ResName=UNL,ResNum=1) -1.62939000 -1.01123000 0.71866500

Fe(PDBName=FE,ResName=UNL,ResNum=1) 0.40308000 1.23477900 0.01949000

Fe(PDBName=FE,ResName=UNL,ResNum=1) -3.88992900 -0.80533300 -0.42279300

Fe(PDBName=FE,ResName=UNL,ResNum=1) -2.37382800 1.41541800 0.33089700

Fe(PDBName=FE,ResName=UNL,ResNum=1) 1.34384300 -1.19131300 -0.72797200

Fe(PDBName=FE,ResName=UNL,ResNum=1) 3.08749000 1.28542400 -0.37145900

O(PDBName=O,ResName=UNL,ResNum=1) 2.33010400 -2.35912000 0.28730900

O(PDBName=O,ResName=UNL,ResNum=1) 2.99512000 -0.42964600 -1.24942700

O(PDBName=O,ResName=UNL,ResNum=1) 1.86901000 0.05943000 0.65998400

O(PDBName=O,ResName=UNL,ResNum=1) -4.11200000 -1.47011700 -1.92322600

O(PDBName=O,ResName=UNL,ResNum=1) -0.04690200 -1.77177500 0.06844900

O(PDBName=O,ResName=UNL,ResNum=1) 1.61676200 1.97238500 -1.12816500

O(PDBName=O,ResName=UNL,ResNum=1) -5.30017100 -1.37834200 0.38278000

O(PDBName=O,ResName=UNL,ResNum=1) 1.29133400 -2.03070300 -2.20747500

O(PDBName=O,ResName=UNL,ResNum=1) 0.21976100 0.17366100 -1.32638300

O(PDBName=O,ResName=UNL,ResNum=1) -2.65041300 -2.18448100 0.01138700

O(PDBName=O,ResName=UNL,ResNum=1) 4.87201900 -1.93292200 -0.16248200

O(PDBName=O,ResName=UNL,ResNum=1) 4.37773900 0.46912400 0.65160900

O(PDBName=O,ResName=UNL,ResNum=1) 3.73999100 -1.41920600 2.15427800

O(PDBName=O,ResName=UNL,ResNum=1) -0.96476600 0.56328300 1.24596500

O(PDBName=O,ResName=UNL,ResNum=1) -1.53144200 -1.72816400 2.25171500

O(PDBName=O,ResName=UNL,ResNum=1) -2.27622000 0.09645900 -0.90411200

O(PDBName=O,ResName=UNL,ResNum=1) 0.81352200 2.14943200 1.30011700

O(PDBName=O,ResName=UNL,ResNum=1) -1.07184600 2.13099700 -0.67179700

O(PDBName=O,ResName=UNL,ResNum=1) -3.36909500 -0.04541900 1.12635100

O(PDBName=O,ResName=UNL,ResNum=1) -4.81698600 0.97720200 -0.79191700

O(PDBName=O,ResName=UNL,ResNum=1) -2.81670900 2.29987000 1.62607500

O(PDBName=O,ResName=UNL,ResNum=1) -4.06222500 1.97120800 -0.66397700

O(PDBName=O,ResName=UNL,ResNum=1) 4.12360700 1.83099000 -1.62979800

O(PDBName=O,ResName=UNL,ResNum=1) 3.20051600 2.56388700 0.71360500

1 8 1.0 18 1.0 19 1.0 20 1.0

2 12 1.0 17 1.0 21 1.0 22 1.0

3 13 1.0 16 1.0 24 1.0 25 1.0

4 11 1.0 14 1.0 17 1.0 23 1.0 26 1.0

5 21 1.0 23 1.0 25 1.0 28 1.0

6 8 1.0 9 1.0 12 1.0 15 1.0 16 1.0

7 9 1.0 13 1.0 19 1.0 30 1.0 31 1.0

8

9

10

11

12

13

14

15

16

17

18

19

20

21

22

23

24

25

26

27 29 1.0

28

29

30

31

- - 1. **Fe7 iron oxide cluster ( *M* = 7)**

----------------------------------------------------------------

# opt=calcfc freq=noraman upm6 geom=connectivity temperature=298

----------------------------------------------------------------

Zero-point correction= 0.082638 (Hartree/Particle)

Thermal correction to Energy= 0.116145

Thermal correction to Enthalpy= 0.117089

Thermal correction to Gibbs Free Energy= 0.021581

Sum of electronic and zero-point Energies= -1.289706

Sum of electronic and thermal Energies= -1.256199

Sum of electronic and thermal Enthalpies= -1.255256

Sum of electronic and thermal Free Energies= -1.350763

-4 7

Fe(PDBName=FE,ResName=UNL,ResNum=1) 3.54411700 -1.08507400 0.51602800

Fe(PDBName=FE,ResName=UNL,ResNum=1) -1.62319800 -1.00202100 0.72434700

Fe(PDBName=FE,ResName=UNL,ResNum=1) 0.40831600 1.24059600 0.00391200

Fe(PDBName=FE,ResName=UNL,ResNum=1) -3.88895800 -0.80099100 -0.43244800

Fe(PDBName=FE,ResName=UNL,ResNum=1) -2.37910700 1.41934100 0.30753300

Fe(PDBName=FE,ResName=UNL,ResNum=1) 1.35399900 -1.18758200 -0.73626600

Fe(PDBName=FE,ResName=UNL,ResNum=1) 3.09540800 1.28522000 -0.35842500

O(PDBName=O,ResName=UNL,ResNum=1) 2.33078200 -2.35685600 0.28215700

O(PDBName=O,ResName=UNL,ResNum=1) 3.01674400 -0.41843800 -1.24222800

O(PDBName=O,ResName=UNL,ResNum=1) 1.86882300 0.06243600 0.65755000

O(PDBName=O,ResName=UNL,ResNum=1) -4.12935300 -1.50789300 -1.89703800

O(PDBName=O,ResName=UNL,ResNum=1) -0.04683900 -1.74716700 0.06321600

O(PDBName=O,ResName=UNL,ResNum=1) 1.62533400 1.98716200 -1.12577000

O(PDBName=O,ResName=UNL,ResNum=1) -5.27047400 -1.44040100 0.41101700

O(PDBName=O,ResName=UNL,ResNum=1) 1.29560300 -2.02709900 -2.21813900

O(PDBName=O,ResName=UNL,ResNum=1) 0.24781100 0.17891500 -1.34918300

O(PDBName=O,ResName=UNL,ResNum=1) -2.62506800 -2.16711000 -0.04001700

O(PDBName=O,ResName=UNL,ResNum=1) 4.87490700 -1.94001200 -0.14721700

O(PDBName=O,ResName=UNL,ResNum=1) 4.37826600 0.46662600 0.67690500

O(PDBName=O,ResName=UNL,ResNum=1) 3.72403300 -1.42976500 2.16130200

O(PDBName=O,ResName=UNL,ResNum=1) -0.97400000 0.59586400 1.23177700

O(PDBName=O,ResName=UNL,ResNum=1) -1.49632600 -1.74768300 2.23917500

O(PDBName=O,ResName=UNL,ResNum=1) -2.26601000 0.09923400 -0.91510100

O(PDBName=O,ResName=UNL,ResNum=1) 0.80944600 2.15561700 1.28991000

O(PDBName=O,ResName=UNL,ResNum=1) -1.06472400 2.10969000 -0.70418000

O(PDBName=O,ResName=UNL,ResNum=1) -3.36313700 -0.06484800 1.11114000

O(PDBName=O,ResName=UNL,ResNum=1) -4.92996700 0.99134700 -0.62265200

O(PDBName=O,ResName=UNL,ResNum=1) -2.88305900 2.28584200 1.59152400

O(PDBName=O,ResName=UNL,ResNum=1) -4.11175000 1.94002100 -0.66055700

O(PDBName=O,ResName=UNL,ResNum=1) 4.13923400 1.84300300 -1.60710300

O(PDBName=O,ResName=UNL,ResNum=1) 3.19034700 2.55567100 0.73330000

1 8 1.0 18 1.0 19 1.0 20 1.0

2 12 1.0 17 1.0 21 1.0 22 1.0

3 13 1.0 16 1.0 24 1.0 25 1.0

4 11 1.0 14 1.0 17 1.0 23 1.0 26 1.0

5 21 1.0 23 1.0 25 1.0 28 1.0

6 8 1.0 9 1.0 12 1.0 15 1.0 16 1.0

7 9 1.0 13 1.0 19 1.0 30 1.0 31 1.0

8

9

10

11

12

13

14

15

16

17

18

19

20

21

22

23

24

25

26

27 29 1.0

28

29

30

31

- - 1. **Fe9 iron oxide cluster ( *M* = 1)**

----------------------------------------------------------------

# opt=calcfc freq=noraman upm6 geom=connectivity temperature=298

----------------------------------------------------------------

Zero-point correction= 0.106817 (Hartree/Particle)

Thermal correction to Energy= 0.146020

Thermal correction to Enthalpy= 0.146964

Thermal correction to Gibbs Free Energy= 0.043385

Sum of electronic and zero-point Energies= -1.115646

Sum of electronic and thermal Energies= -1.076443

Sum of electronic and thermal Enthalpies= -1.075499

Sum of electronic and thermal Free Energies= -1.179078

-6 1

Fe(PDBName=FE,ResName=UNL,ResNum=1) -2.86807800 -1.42797100 -1.15945100

Fe(PDBName=FE,ResName=UNL,ResNum=1) 0.28223600 -0.19469900 -1.10046000

Fe(PDBName=FE,ResName=UNL,ResNum=1) 1.68132000 -1.34744700 0.98224600

Fe(PDBName=FE,ResName=UNL,ResNum=1) 0.39273400 1.81325800 0.42335700

Fe(PDBName=FE,ResName=UNL,ResNum=1) 3.74130600 -1.18204600 -0.50858500

Fe(PDBName=FE,ResName=UNL,ResNum=1) -2.24604800 1.11388000 1.84309700

Fe(PDBName=FE,ResName=UNL,ResNum=1) 3.11683200 1.52374500 -0.12092100

Fe(PDBName=FE,ResName=UNL,ResNum=1) -1.45941200 -1.60915900 1.08932000

Fe(PDBName=FE,ResName=UNL,ResNum=1) -2.76575200 1.28340900 -0.97640200

O(PDBName=O,ResName=UNL,ResNum=1) -2.85876100 -2.52655700 0.25291200

O(PDBName=O,ResName=UNL,ResNum=1) -2.64432200 -0.14906400 0.35933900

O(PDBName=O,ResName=UNL,ResNum=1) -0.87530800 1.34434400 -0.89050600

O(PDBName=O,ResName=UNL,ResNum=1) 3.34096700 -2.35197900 0.76725200

O(PDBName=O,ResName=UNL,ResNum=1) -1.05058700 -1.49240400 -0.77692200

O(PDBName=O,ResName=UNL,ResNum=1) 0.17436000 -2.39172900 0.94154000

O(PDBName=O,ResName=UNL,ResNum=1) -2.71787100 2.25634400 0.53943700

O(PDBName=O,ResName=UNL,ResNum=1) 3.83122400 -2.08101300 -1.94453800

O(PDBName=O,ResName=UNL,ResNum=1) -2.11803100 -2.51148500 2.38214500

O(PDBName=O,ResName=UNL,ResNum=1) 0.17085500 -0.05008800 0.72156600

O(PDBName=O,ResName=UNL,ResNum=1) 1.77551700 -1.40200000 -0.76710500

O(PDBName=O,ResName=UNL,ResNum=1) -4.51473000 -1.28139100 -1.08359800

O(PDBName=O,ResName=UNL,ResNum=1) -2.50674000 -0.01083200 -2.14419200

O(PDBName=O,ResName=UNL,ResNum=1) -2.56888200 -2.45329100 -2.49073800

O(PDBName=O,ResName=UNL,ResNum=1) -1.46811000 -0.30997700 2.41563400

O(PDBName=O,ResName=UNL,ResNum=1) 1.45692300 1.29606900 -0.97866400

O(PDBName=O,ResName=UNL,ResNum=1) 0.41163000 -0.19749200 -2.67957900

O(PDBName=O,ResName=UNL,ResNum=1) 1.73366700 -1.16226700 2.58277400

O(PDBName=O,ResName=UNL,ResNum=1) 3.14086200 -0.04478500 0.86101900

O(PDBName=O,ResName=UNL,ResNum=1) 0.37958500 3.37103600 -0.04223600

O(PDBName=O,ResName=UNL,ResNum=1) 1.93468800 1.92907200 1.26970900

O(PDBName=O,ResName=UNL,ResNum=1) 3.77658100 0.35487900 -1.38173800

O(PDBName=O,ResName=UNL,ResNum=1) 5.34585700 -1.06894300 -0.00384000

O(PDBName=O,ResName=UNL,ResNum=1) -3.80203800 0.87543600 2.31357100

O(PDBName=O,ResName=UNL,ResNum=1) -0.70643100 1.87553100 1.91884500

O(PDBName=O,ResName=UNL,ResNum=1) 3.34560800 2.69689600 -1.34093900

O(PDBName=O,ResName=UNL,ResNum=1) 4.43035200 1.96596500 0.84869600

O(PDBName=O,ResName=UNL,ResNum=1) -4.41358000 1.17162000 -0.95257500

O(PDBName=O,ResName=UNL,ResNum=1) -2.59747900 2.43595300 -2.23192800

1 10 1.0 14 1.0 21 1.0 22 1.0 23 1.0

2 14 1.0 19 1.0 25 1.0 26 1.0

3 15 1.0 20 1.0 27 1.0

4 12 1.0 19 1.0 25 1.0 29 1.0 30 1.0 34 1.0

5 13 1.0 17 1.0 28 1.0 31 1.0 32 1.0

6 16 1.0 24 1.0 33 1.0 34 1.0

7 25 1.0 28 1.0 30 1.0 31 1.0 35 1.0 36 1.0

8 10 1.0 14 1.0 15 1.0 18 1.0 24 1.0

9 12 1.0 16 1.0 22 1.0 37 1.0 38 1.0

10

11

12

13

14

15

16

17

18

19

20

21

22

23

24

25

26

27

28

29

30

31

32

33

34

35

36

37

38

- - 1. **Fe9 iron oxide cluster ( *M* = 5)**

----------------------------------------------------------------

# opt=calcfc freq=noraman upm6 geom=connectivity temperature=298

----------------------------------------------------------------

Zero-point correction= 0.102530 (Hartree/Particle)

Thermal correction to Energy= 0.142980

Thermal correction to Enthalpy= 0.143924

Thermal correction to Gibbs Free Energy= 0.036599

Sum of electronic and zero-point Energies= -1.138702

Sum of electronic and thermal Energies= -1.098252

Sum of electronic and thermal Enthalpies= -1.097308

Sum of electronic and thermal Free Energies= -1.204633

-6 5

Fe(PDBName=FE,ResName=UNL,ResNum=1) -2.72534000 -1.48871100 -1.13151700

Fe(PDBName=FE,ResName=UNL,ResNum=1) 0.34616500 -0.09404400 -1.18416100

Fe(PDBName=FE,ResName=UNL,ResNum=1) 1.62835300 -1.35317400 0.95876200

Fe(PDBName=FE,ResName=UNL,ResNum=1) 0.32422200 1.88566200 0.38189800

Fe(PDBName=FE,ResName=UNL,ResNum=1) 3.71512300 -1.12583100 -0.49387100

Fe(PDBName=FE,ResName=UNL,ResNum=1) -2.36567000 1.06105200 1.82374400

Fe(PDBName=FE,ResName=UNL,ResNum=1) 3.06580500 1.53182500 -0.06577500

Fe(PDBName=FE,ResName=UNL,ResNum=1) -1.31821000 -1.55560300 1.04967200

Fe(PDBName=FE,ResName=UNL,ResNum=1) -2.86928100 1.23993800 -0.91625700

O(PDBName=O,ResName=UNL,ResNum=1) -2.38994100 -2.73219400 0.26793500

O(PDBName=O,ResName=UNL,ResNum=1) -2.74069100 -0.24475700 0.32750600

O(PDBName=O,ResName=UNL,ResNum=1) -0.93639900 1.36663100 -0.81916000

O(PDBName=O,ResName=UNL,ResNum=1) 3.17260200 -2.40708000 0.68844500

O(PDBName=O,ResName=UNL,ResNum=1) -0.90865600 -1.39774000 -0.84605400

O(PDBName=O,ResName=UNL,ResNum=1) 0.34136100 -2.52078900 0.85120500

O(PDBName=O,ResName=UNL,ResNum=1) -3.10875900 2.17686700 0.65223100

O(PDBName=O,ResName=UNL,ResNum=1) 4.03329800 -2.01868500 -1.89871600

O(PDBName=O,ResName=UNL,ResNum=1) -1.82111800 -2.51775300 2.49017600

O(PDBName=O,ResName=UNL,ResNum=1) 0.25896700 -0.04674400 0.67012000

O(PDBName=O,ResName=UNL,ResNum=1) 1.80184800 -1.30935200 -0.84976600

O(PDBName=O,ResName=UNL,ResNum=1) -4.39541300 -1.67079400 -1.03686400

O(PDBName=O,ResName=UNL,ResNum=1) -2.63373200 -0.06119200 -2.15829800

O(PDBName=O,ResName=UNL,ResNum=1) -2.46201500 -2.50514600 -2.49820800

O(PDBName=O,ResName=UNL,ResNum=1) -1.27896300 -0.27606200 2.29538300

O(PDBName=O,ResName=UNL,ResNum=1) 1.49477700 1.35454600 -1.00368400

O(PDBName=O,ResName=UNL,ResNum=1) 0.48950200 0.00038400 -2.80483800

O(PDBName=O,ResName=UNL,ResNum=1) 1.68064200 -1.20522200 2.60141800

O(PDBName=O,ResName=UNL,ResNum=1) 3.06452000 -0.00848200 0.88353500

O(PDBName=O,ResName=UNL,ResNum=1) 0.29446700 3.50145000 -0.12501000

O(PDBName=O,ResName=UNL,ResNum=1) 1.79934400 2.08021200 1.26618100

O(PDBName=O,ResName=UNL,ResNum=1) 3.86946800 0.39692200 -1.35192000

O(PDBName=O,ResName=UNL,ResNum=1) 5.30360000 -1.04713000 0.16513300

O(PDBName=O,ResName=UNL,ResNum=1) -3.80330800 0.50331300 2.40914600

O(PDBName=O,ResName=UNL,ResNum=1) -0.94236100 2.00433900 1.79865000

O(PDBName=O,ResName=UNL,ResNum=1) 3.38020500 2.74594600 -1.20065500

O(PDBName=O,ResName=UNL,ResNum=1) 4.39490300 2.01019400 1.00210500

O(PDBName=O,ResName=UNL,ResNum=1) -4.56785900 1.08740200 -1.01634000

O(PDBName=O,ResName=UNL,ResNum=1) -2.74408400 2.41229800 -2.13275800

1 10 1.0 11 1.0 14 1.0 21 1.0 22 1.0 23 1.0

2 14 1.0 19 1.0 20 1.0 25 1.0 26 1.0

3 13 1.0 15 1.0 19 1.0 20 1.0 27 1.0

4 12 1.0 25 1.0 29 1.0 30 1.0 34 1.0

5 13 1.0 17 1.0 28 1.0 31 1.0 32 1.0

6 16 1.0 24 1.0 33 1.0 34 1.0

7 25 1.0 28 1.0 30 1.0 31 1.0 35 1.0 36 1.0

8 10 1.0 18 1.0 24 1.0

9 16 1.0 22 1.0 37 1.0 38 1.0

10

11

12

13

14

15

16

17

18

19

20

21

22

23

24

25

26

27

28

29

30

31

32

33

34

35

36

37

38

- - 1. **Fe9 iron oxide cluster ( *M* = 7)**

----------------------------------------------------------------

# opt=calcfc freq=noraman upm6 geom=connectivity temperature=298

----------------------------------------------------------------

Zero-point correction= 0.102479 (Hartree/Particle)

Thermal correction to Energy= 0.142864

Thermal correction to Enthalpy= 0.143807

Thermal correction to Gibbs Free Energy= 0.036733

Sum of electronic and zero-point Energies= -1.135912

Sum of electronic and thermal Energies= -1.095527

Sum of electronic and thermal Enthalpies= -1.094584

Sum of electronic and thermal Free Energies= -1.201658

-6 7

Fe(PDBName=FE,ResName=UNL,ResNum=1) -2.75748300 -1.50424900 -1.07738000

Fe(PDBName=FE,ResName=UNL,ResNum=1) 0.35751700 -0.18971900 -1.17351700

Fe(PDBName=FE,ResName=UNL,ResNum=1) 1.63302800 -1.34824100 1.01416000

Fe(PDBName=FE,ResName=UNL,ResNum=1) 0.30369000 1.82690300 0.29638600

Fe(PDBName=FE,ResName=UNL,ResNum=1) 3.70071300 -1.11496200 -0.47242800

Fe(PDBName=FE,ResName=UNL,ResNum=1) -2.28888800 1.16386300 1.79324300

Fe(PDBName=FE,ResName=UNL,ResNum=1) 3.04836300 1.55112500 -0.10395200

Fe(PDBName=FE,ResName=UNL,ResNum=1) -1.28030600 -1.51000000 1.04873100

Fe(PDBName=FE,ResName=UNL,ResNum=1) -2.85457100 1.20697200 -0.92358900

O(PDBName=O,ResName=UNL,ResNum=1) -2.35332900 -2.72611000 0.32061300

O(PDBName=O,ResName=UNL,ResNum=1) -2.73867500 -0.21348300 0.33033700

O(PDBName=O,ResName=UNL,ResNum=1) -0.93166700 1.24896100 -0.91042200

O(PDBName=O,ResName=UNL,ResNum=1) 3.14188500 -2.40588700 0.73719900

O(PDBName=O,ResName=UNL,ResNum=1) -0.91643700 -1.43944200 -0.86402100

O(PDBName=O,ResName=UNL,ResNum=1) 0.35570100 -2.51035700 0.89378700

O(PDBName=O,ResName=UNL,ResNum=1) -2.98128100 2.26764100 0.61801000

O(PDBName=O,ResName=UNL,ResNum=1) 4.02510000 -1.95447900 -1.90710000

O(PDBName=O,ResName=UNL,ResNum=1) -1.85928300 -2.43614200 2.55556900

O(PDBName=O,ResName=UNL,ResNum=1) 0.24914500 -0.08365300 0.69054300

O(PDBName=O,ResName=UNL,ResNum=1) 1.79465700 -1.33427300 -0.83201700

O(PDBName=O,ResName=UNL,ResNum=1) -4.44851600 -1.62668600 -0.92083400

O(PDBName=O,ResName=UNL,ResNum=1) -2.71319600 -0.10916900 -2.16434000

O(PDBName=O,ResName=UNL,ResNum=1) -2.57810300 -2.54068300 -2.41122300

O(PDBName=O,ResName=UNL,ResNum=1) -1.28941700 -0.21914800 2.30092900

O(PDBName=O,ResName=UNL,ResNum=1) 1.51941200 1.27129800 -1.05102600

O(PDBName=O,ResName=UNL,ResNum=1) 0.52163900 -0.25011600 -2.81432500

O(PDBName=O,ResName=UNL,ResNum=1) 1.62575100 -1.05011100 2.63237100

O(PDBName=O,ResName=UNL,ResNum=1) 3.02821700 0.00188100 0.88151000

O(PDBName=O,ResName=UNL,ResNum=1) 0.25239300 3.42959600 -0.26796200

O(PDBName=O,ResName=UNL,ResNum=1) 1.73847000 2.10007400 1.21383400

O(PDBName=O,ResName=UNL,ResNum=1) 3.88066100 0.40326800 -1.33329000

O(PDBName=O,ResName=UNL,ResNum=1) 5.27765000 -1.01219700 0.21311100

O(PDBName=O,ResName=UNL,ResNum=1) -3.73477900 0.64666400 2.40297100

O(PDBName=O,ResName=UNL,ResNum=1) -0.83889200 2.05893500 1.78279200

O(PDBName=O,ResName=UNL,ResNum=1) 3.36983300 2.74824000 -1.25933900

O(PDBName=O,ResName=UNL,ResNum=1) 4.35758100 2.00353600 0.96760900

O(PDBName=O,ResName=UNL,ResNum=1) -4.55960000 1.11206700 -0.94589000

O(PDBName=O,ResName=UNL,ResNum=1) -2.74662300 2.35427700 -2.16476800

1 10 1.0 11 1.0 14 1.0 21 1.0 22 1.0 23 1.0

2 14 1.0 19 1.0 20 1.0 25 1.0 26 1.0

3 13 1.0 15 1.0 19 1.0 20 1.0 27 1.0

4 12 1.0 25 1.0 29 1.0 30 1.0 34 1.0

5 13 1.0 17 1.0 28 1.0 31 1.0 32 1.0

6 16 1.0 24 1.0 33 1.0 34 1.0

7 25 1.0 28 1.0 31 1.0 35 1.0 36 1.0

8 10 1.0 15 1.0 18 1.0 24 1.0

9 11 1.0 12 1.0 16 1.0 22 1.0 37 1.0 38 1.0

10

11

12

13

14

15

16

17

18

19

20

21

22

23

24

25

26

27

28

29

30

31

32

33

34

35

36

37

38

**3. Antibacterial Analyses, Data**


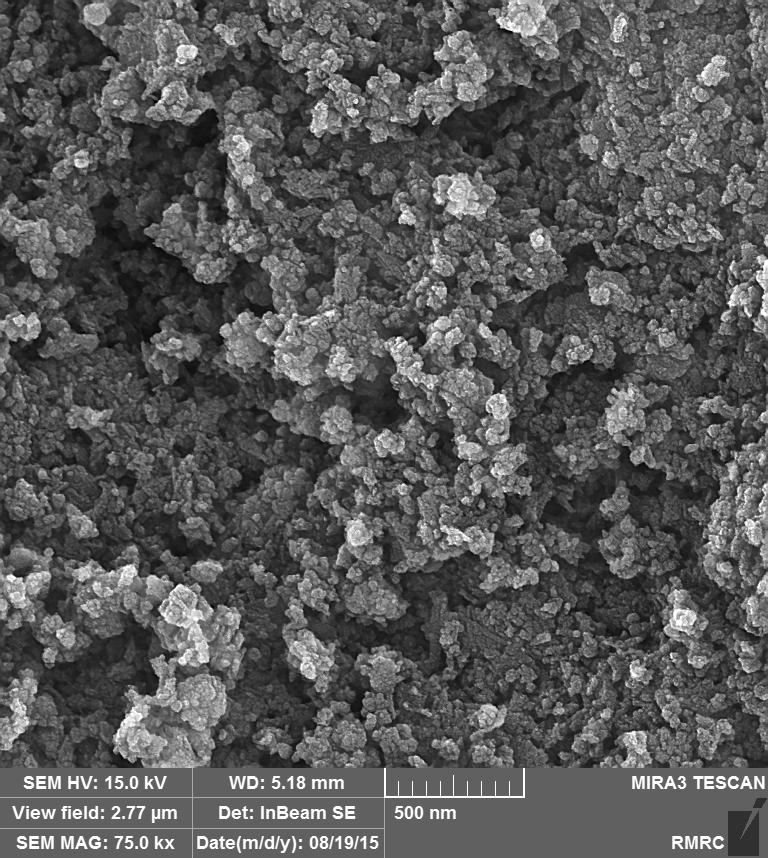


**Figure S2.** FESEM image of the nanoparticles deposited on a silicon wafer. This is a zoom-out of the image previously published in reference 10. Note the relative uniformity of the nanoparticles on the surface.

**Table S1.** Antibacterial raw data related to Minimum Inhibitory Concentration (μg/mL) of the 19 nm nanomagnetite and two positive controls. Each entry represents a separate experiment, and the determined MIC based on serial dilution.

| S. aureus | E. Coli | No | Nanomag. |
| --- | --- | --- | --- |
| 4.06 | 4.06 | 1 |  |
| 2.03 | 2.03 | 2 |  |
| 2.03 | 4.06 | 3 |  |
| 1.01 | 2.03 | 4 |  |
| 1.01 | 2.03 | 5 |  |
| 1.01 | 2.03 | 6 |  |
| 4.06 | 4.06 | 7 |  |
| 1.01 | 2.03 | 8 |  |
| 2.0275 | 2.79125 | Average |  |
| 1.331130025 | 1.050624814 | SD |  |
| 8 | 8 | Number |  |
| 0.4706255336 | 0.3714519653 | SE |  |
|  |  |  |  |
|  |  |  |  |
|  |  |  |  |
| S. areus | E. coli | No. | Amp. |
| 0.625 | 0.625 | 1 |  |
| 1.25 | 0.625 | 2 |  |
| 0.625 | 0.625 | 3 |  |
| 1.25 | 1.25 | 4 |  |
| 0.625 | 0.625 | 5 |  |
| 0.625 | 1.25 | 6 |  |
| 0.625 | 0.625 | 7 |  |
| 1.25 | 0.625 | 8 |  |
| 0.859375 | 0.78125 | Average |  |
| 0.323468231 | 0.289318781 | SD |  |
| 0.11 | 0.09 | SE |  |
|  |  |  |  |
|  |  |  |  |
|  |  |  |  |
| S. areus | E. coli | No. | Chloram. |
| 0.312 | 0.312 | 1 |  |
| 0.312 | 0.312 | 2 |  |
| 0.625 | 0.312 | 3 |  |
| 0.625 | 0.312 | 4 |  |
| 0.312 | 0.312 | 5 |  |
| 0.625 | 0.625 | 6 |  |
| 0.625 | 0.625 | 7 |  |
| 0.312 | 0.625 | 8 |  |
| 0.4685 | 0.429375 | Average |  |
| 0.1673055374 | 0.1619928901 | SD |  |
| 0.05 | 0.05 | SE |  |

**4. Liver-related Enzyme Activity.**

All measurements are in units of activity per litre for ALT and AST.

**Table S2.** ALT levels in serum after 14 days of daily exposure to the treatment at the given concentration (for details, see SI section 1.4)

|  |  | ALT |
| --- | --- | --- |
|  |  |  |
| control | 1 | 54 |
|  | 2 | 64 |
|  | 3 | 80 |
|  | 4 | 64 |
|  | 5 | 58 |
|  | 6 | 48 |
|  | 7 | 61 |
|  | 8 | 63 |
|  | 9 | 50 |
|  | 10 | 63 |
|  | average | 60.5 |
|  | sd | 9.021579 |
| 10 ug/mL |  |  |
|  | 1 | 76 |
|  | 2 | 89 |
|  | 3 | 98 |
|  | 4 | 56 |
|  | 5 | 65 |
|  | 6 | 61 |
|  | 7 | 75 |
|  | 8 | 80 |
|  | 9 | 48 |
|  | 10 | 51 |
|  | average | 69.9 |
|  | sd | 16.50892 |
|  |  |  |
| 100 ug/mL | 1 | 110 |
|  | 2 | 99 |
|  | 3 | 108 |
|  | 4 | 80 |
|  | 5 | 76 |
|  | 6 | 90 |
|  | 7 | 120 |
|  | 8 | 110 |
|  | 9 | 85 |
|  | 10 | 140 |
|  |  | 101.8 |
|  |  | 19.8147 |
|  |  |  |
| 1000 ug/mL | 1 | 120 |
|  | 2 | 112 |
|  | 3 | 101 |
|  | 4 | 98 |
|  | 5 | 102 |
|  | 6 | 89 |
|  | 7 | 123 |
|  | 8 | 130 |
|  | 9 | 120 |
|  |  |  |
|  |  | 110.5556 |
|  |  | 13.69408 |

| **Table S3.** ANOVA of ALT levels. | | | | | |
| --- | --- | --- | --- | --- | --- |
|  | | | | | |
|  | Sum of Squares | df | Mean Square | F | Sig. |
| Between Groups | 16982.675 | 3 | 5660.892 | 24.106 | .000 |
| Within Groups | 8219.222 | 35 | 234.835 |  |  |
| Total | 25201.897 | 38 |  |  |  |

| **Table S4.** Multiple Comparisons, Tukey HSD | | | | | | |
| --- | --- | --- | --- | --- | --- | --- |
|  | | | | | | |
| (I) VAR00001 | (J) VAR00001 | Mean Difference (I-J) | Std. Error | Sig. | 95% Confidence Interval | |
|  |  |  |  |  | Lower Bound | Upper Bound |
| 1.00 | 2.00 | -9.40000 | 6.85325 | .525 | -27.8825 | 9.0825 |
|  | 3.00 | -41.30000^*^ | 6.85325 | .000 | -59.7825 | -22.8175 |
|  | 4.00 | -50.05556^*^ | 7.04104 | .000 | -69.0446 | -31.0666 |
| 2.00 | 1.00 | 9.40000 | 6.85325 | .525 | -9.0825 | 27.8825 |
|  | 3.00 | -31.90000^*^ | 6.85325 | .000 | -50.3825 | -13.4175 |
|  | 4.00 | -40.65556^*^ | 7.04104 | .000 | -59.6446 | -21.6666 |
| 3.00 | 1.00 | 41.30000^*^ | 6.85325 | .000 | 22.8175 | 59.7825 |
|  | 2.00 | 31.90000^*^ | 6.85325 | .000 | 13.4175 | 50.3825 |
|  | 4.00 | -8.75556 | 7.04104 | .604 | -27.7446 | 10.2334 |
| 4.00 | 1.00 | 50.05556^*^ | 7.04104 | .000 | 31.0666 | 69.0446 |
|  | 2.00 | 40.65556^*^ | 7.04104 | .000 | 21.6666 | 59.6446 |
|  | 3.00 | 8.75556 | 7.04104 | .604 | -10.2334 | 27.7446 |
| *. The mean difference is significant at the 0.05 level. | | | | | | |

**Table S5.** AST levels in serum after 14 days of daily exposure to the treatment at the given concentration (for details, see SI section 1.4)

| \| control \| 1 \| 43 \| \| --- \| --- \| --- \| \|  \| 2 \| 41 \| \|  \| 3 \| 57 \| \|  \| 4 \| 41 \| \|  \| 5 \| 32 \| \|  \| 6 \| 25 \| \|  \| 7 \| 31 \| \|  \| 8 \| 43 \| \|  \| 9 \| 50 \| \|  \| 10 \| 28 \| \|  \| average \| 39.1 \| \|  \| sd \| 10.08244 \| \| 10 ug/mL \|  \|  \| \|  \| 1 \| 54 \| \|  \| 2 \| 43 \| \|  \| 3 \| 34 \| \|  \| 4 \| 60 \| \|  \| 5 \| 42 \| \|  \| 6 \| 50 \| \|  \| 7 \| 43 \| \|  \| 8 \| 55 \| \|  \| 9 \| 45 \| \|  \| 10 \| 47 \| \|  \| average \| 47.3 \| \|  \| sd \| 7.6019 \| \|  \|  \|  \| \| 100 ug/mL \| 1 \| 72 \| \|  \| 2 \| 70 \| \|  \| 3 \| 38 \| \|  \| 4 \| 78 \| \|  \| 5 \| 86 \| \|  \| 6 \| 80 \| \|  \| 7 \| 73 \| \|  \| 8 \| 69 \| \|  \| 9 \| 65 \| \|  \| 10 \| 80 \| \|  \| average \| 71.1 \| \|  \| sd \| 13.21 \| \|  \|  \|  \| \| 1000 ug/mL \| 1 \| 80 \| \|  \| 2 \| 64 \| \|  \| 3 \| 87 \| \|  \| 4 \| 72 \| \|  \| 5 \| 59 \| \|  \| 6 \| 58 \| \|  \| 7 \| 62 \| \|  \| 8 \| 83 \| \|  \| 9 \| 60 \| \|  \| Average \| 69.44 \| \|  \| sd \| 11.31 \| |
| --- | --- | --- | --- | --- | --- | --- | --- | --- | --- | --- | --- | --- | --- | --- | --- | --- | --- | --- | --- | --- | --- | --- | --- | --- | --- | --- | --- | --- | --- | --- | --- | --- | --- | --- | --- | --- | --- | --- | --- | --- | --- | --- | --- | --- | --- | --- | --- | --- | --- | --- | --- | --- | --- | --- | --- | --- | --- | --- | --- | --- | --- | --- | --- | --- | --- | --- | --- | --- | --- | --- | --- | --- | --- | --- | --- | --- | --- | --- | --- | --- | --- | --- | --- | --- | --- | --- | --- | --- | --- | --- | --- | --- | --- | --- | --- | --- | --- | --- | --- | --- | --- | --- | --- | --- | --- | --- | --- | --- | --- | --- | --- | --- | --- | --- | --- | --- | --- | --- | --- | --- | --- | --- | --- | --- | --- | --- | --- | --- | --- | --- | --- | --- | --- | --- | --- | --- | --- | --- | --- | --- | --- | --- | --- | --- | --- | --- | --- | --- | --- | --- |

| **Table S6.** Serum AST ANOVA | | | | | |
| --- | --- | --- | --- | --- | --- |
| VAR00002 | | | | | |
|  | Sum of Squares | df | Mean Square | F | Sig. |
| Between Groups | 26216.409 | 3 | 8738.803 | 71.515 | .000 |
| Within Groups | 4276.822 | 35 | 122.195 |  |  |
| Total | 30493.231 | 38 |  |  |  |

| **Table S7.** Multiple Comparisons, Tukey HSD of Serum AST | | | | | | |
| --- | --- | --- | --- | --- | --- | --- |
| Dependent Variable: VAR00002  Tukey HSD | | | | | | |
| (I) VAR00001 | (J) VAR00001 | Mean Difference (I-J) | Std. Error | Sig. | 95% Confidence Interval | |
|  |  |  |  |  | Lower Bound | Upper Bound |
| 1.00 | 2.00 | -8.20000 | 4.94358 | .360 | -21.5324 | 5.1324 |
|  | 3.00 | -18.10000^*^ | 4.94358 | .004 | -31.4324 | -4.7676 |
|  | 4.00 | -68.34444^*^ | 5.07905 | .000 | -82.0421 | -54.6468 |
| 2.00 | 1.00 | 8.20000 | 4.94358 | .360 | -5.1324 | 21.5324 |
|  | 3.00 | -9.90000 | 4.94358 | .206 | -23.2324 | 3.4324 |
|  | 4.00 | -60.14444^*^ | 5.07905 | .000 | -73.8421 | -46.4468 |
| 3.00 | 1.00 | 18.10000^*^ | 4.94358 | .004 | 4.7676 | 31.4324 |
|  | 2.00 | 9.90000 | 4.94358 | .206 | -3.4324 | 23.2324 |
|  | 4.00 | -50.24444^*^ | 5.07905 | .000 | -63.9421 | -36.5468 |
| 4.00 | 1.00 | 68.34444^*^ | 5.07905 | .000 | 54.6468 | 82.0421 |
|  | 2.00 | 60.14444^*^ | 5.07905 | .000 | 46.4468 | 73.8421 |
|  | 3.00 | 50.24444^*^ | 5.07905 | .000 | 36.5468 | 63.9421 |
| *. The mean difference is significant at the 0.05 level. | | | | | | |

**5. Catalase and oxidative damage data**

All catalase measurements are in units/mL. All MDA data are in nmol/ml.

**Table S8.**  Serum catalase levels.

|  |  | CATALASE |
| --- | --- | --- |
| control | 1 | 7.9 |
|  | 2 | 9.0 |
|  | 3 | 6 |
|  | 4 | 9.1 |
|  | 5 | 7.3 |
|  | 6 | 9.2 |
|  | 7 | 9.5 |
|  | 8 | 9 |
|  | 9 | 8.0 |
|  | 10 | 6 |
|  | average | 8.1 |
|  | sd | 1.30384 |
| 10 ug/mL |  |  |
|  | 1 | 9 |
|  | 2 | 7 |
|  | 3 | 9 |
|  | 4 | 8 |
|  | 5 | 7.4 |
|  | 6 | 8.7 |
|  | 7 | 9.7 |
|  | 8 | 9 |
|  | 9 | 8 |
|  | 10 | 9.1 |
|  | average | 8.49 |
|  | sd | 0.853034 |
|  |  |  |
| 100 ug/mL | |  |
|  | 1 | 6.3 |
|  | 2 | 7.8 |
|  | 3 | 5.1 |
|  | 4 | 5.8 |
|  | 5 | 4.5 |
|  | 6 | 4.6 |
|  | 7 | 6.9 |
|  | 8 | 8 |
|  | 9 | 7 |
|  | 10 | 8 |
|  |  | 6.4 |
|  |  | 1.358103 |
| 1000 ug/mL | |  |
|  | 1 | 5.3 |
|  | 2 | 4.8 |
|  | 3 | 4.7 |
|  | 4 | 4.9 |
|  | 5 | 4.2 |
|  | 6 | 5.9 |
|  | 7 | 5.3 |
|  | 8 | 6.0 |
|  | 9 | 7.5 |
| average |  | 5.4 |
| sd |  | 0.969473 |

| **Table S9.** Multiple Comparisons, Tukey HSD of Serum catalase | | | | | | |
| --- | --- | --- | --- | --- | --- | --- |
| Dependent Variable: VAR00002  Tukey HSD | | | | | | |
| (I) VAR00001 | (J) VAR00001 | Mean Difference (I-J) | Std. Error | Sig. | 95% Confidence Interval | |
|  |  |  |  |  | Lower Bound | Upper Bound |
| 1.00 | 2.00 | -.39000 | .51286 | .872 | -1.7731 | .9931 |
|  | 3.00 | 1.70000^*^ | .51286 | .011 | .3169 | 3.0831 |
|  | 4.00 | 2.70000^*^ | .52691 | .000 | 1.2790 | 4.1210 |
| 2.00 | 1.00 | .39000 | .51286 | .872 | -.9931 | 1.7731 |
|  | 3.00 | 2.09000^*^ | .51286 | .001 | .7069 | 3.4731 |
|  | 4.00 | 3.09000^*^ | .52691 | .000 | 1.6690 | 4.5110 |
| 3.00 | 1.00 | -1.70000^*^ | .51286 | .011 | -3.0831 | -.3169 |
|  | 2.00 | -2.09000^*^ | .51286 | .001 | -3.4731 | -.7069 |
|  | 4.00 | 1.00000 | .52691 | .247 | -.4210 | 2.4210 |
| 4.00 | 1.00 | -2.70000^*^ | .52691 | .000 | -4.1210 | -1.2790 |
|  | 2.00 | -3.09000^*^ | .52691 | .000 | -4.5110 | -1.6690 |
|  | 3.00 | -1.00000 | .52691 | .247 | -2.4210 | .4210 |
| *. The mean difference is significant at the 0.05 level. | | | | | | |

**Table S10.** MDA levels after 14-day treatment.

| control | 1 | 0.9 |
| --- | --- | --- |
|  | 2 | 1.1 |
|  | 3 | 1.4 |
|  | 4 | 1.3 |
|  | 5 | 1.7 |
|  | 6 | 1.9 |
|  | 7 | 1.9 |
|  | 8 | 2 |
|  | 9 | 1.0 |
|  | 10 | 1.8 |
|  | average | 1.5 |
|  | sd | 0.408862 |
| 10 ug/mL |  |  |
|  | 1 | 2 |
|  | 2 | 1.9 |
|  | 3 | 1.8 |
|  | 4 | 2 |
|  | 5 | 1.48 |
|  | 6 | 1.74 |
|  | 7 | 1.5 |
|  | 8 | 1.3 |
|  | 9 | 1.5 |
|  | 10 | 1.8 |
|  | average | 1.702 |
|  | sd | 0.242661 |
|  |  |  |
| 100 ug/mL | |  |
|  | 1 | 2.7 |
|  | 2 | 2 |
|  | 3 | 2.4 |
|  | 4 | 1.1 |
|  | 5 | 2.166667 |
|  | 6 | 2.2 |
|  | 7 | 2 |
|  | 8 | 1.9 |
|  | 9 | 1.1 |
|  | 10 | 1.7 |
|  |  | 1.926667 |
|  |  | 0.514674 |
| 1000 ug/mL | |  |
|  | 1 | 3.3 |
|  | 2 | 2.0 |
|  | 3 | 2.7 |
|  | 4 | 2.9 |
|  | 5 | 2.2 |
|  | 6 | 4.0 |
|  | 7 | 4.0 |
|  | 8 | 3.0 |
|  | 9 | 2.3 |
| average |  | 2.9 |
| sd |  | 0.730795 |

| **Table S11.** Multiple Comparisons, Tukey HSD of Serum MDA | | | | | | |
| --- | --- | --- | --- | --- | --- | --- |
| Dependent Variable: VAR00002  Tukey HSD | | | | | | |
| (I) VAR00001 | (J) VAR00001 | Mean Difference (I-J) | Std. Error | Sig. | 95% Confidence Interval | |
|  |  |  |  |  | Lower Bound | Upper Bound |
| 1.00 | 2.00 | -.20200 | .22315 | .802 | -.8038 | .3998 |
|  | 3.00 | -.42667 | .22315 | .242 | -1.0285 | .1751 |
|  | 4.00 | -1.43333^*^ | .22926 | .000 | -2.0516 | -.8150 |
| 2.00 | 1.00 | .20200 | .22315 | .802 | -.3998 | .8038 |
|  | 3.00 | -.22467 | .22315 | .746 | -.8265 | .3771 |
|  | 4.00 | -1.23133^*^ | .22926 | .000 | -1.8496 | -.6130 |
| 3.00 | 1.00 | .42667 | .22315 | .242 | -.1751 | 1.0285 |
|  | 2.00 | .22467 | .22315 | .746 | -.3771 | .8265 |
|  | 4.00 | -1.00667^*^ | .22926 | .001 | -1.6250 | -.3884 |
| 4.00 | 1.00 | 1.43333^*^ | .22926 | .000 | .8150 | 2.0516 |
|  | 2.00 | 1.23133^*^ | .22926 | .000 | .6130 | 1.8496 |
|  | 3.00 | 1.00667^*^ | .22926 | .001 | .3884 | 1.6250 |
| *. The mean difference is significant at the 0.05 level. | | | | | | |

**6. Markers of Kidney Damage**

**All data is expressed in terms of mg of analyte per dl of serum.**

**Table S12.** Serum creatinine levels.

| control | 1 | 0.5 |
| --- | --- | --- |
|  | 2 | 0.9 |
|  | 3 | 0.7 |
|  | 4 | 0.68 |
|  | 5 | 0.6 |
|  | 6 | 0.6 |
|  | 7 | 0.7 |
|  | 8 | 0.72 |
|  | 9 | 0.70 |
|  | 10 | 0.71 |
|  | average | 0.68 |
|  | sd | 0.092229 |
| 10 ug/mL |  |  |
|  | 1 | 0.80 |
|  | 2 | 0.70 |
|  | 3 | 0.76 |
|  | 4 | 0.60 |
|  | 5 | 0.60 |
|  | 6 | 0.70 |
|  | 7 | 0.50 |
|  | 8 | 0.52 |
|  | 9 | 0.80 |
|  | 10 | 0.65 |
|  | average | 0.662947 |
|  | sd | 0.108539 |
|  |  |  |
| 100 ug/mL | 1 | 0.69 |
|  | 2 | 0.90 |
|  | 3 | 1.03 |
|  | 4 | 0.91 |
|  | 5 | 0.90 |
|  | 6 | 1.01 |
|  | 7 | 0.98 |
|  | 8 | 0.7 |
|  | 9 | 1.60 |
|  | 10 | 1.00 |
|  | average | 0.97 |
|  | sd | 0.250611 |
|  |  |  |
| 1000 ug/mL | 1 | 1 |
|  | 2 | 1.1 |
|  | 3 | 1.2 |
|  | 4 | 1.1 |
|  | 5 | 1.3 |
|  | 6 | 1 |
|  | 7 | 1.057143 |
|  | 8 | 1.4 |
|  | 9 | 1 |
|  |  | 1.128571 |

**Table S13.** Multiple Comparisons, Tukey HSD of Serum creatinine

| Dependent Variable: VAR00002  Tukey HSD | | | | | | |
| --- | --- | --- | --- | --- | --- | --- |
| (I) VAR00001 | (J) VAR00001 | Mean Difference (I-J) | Std. Error | Sig. | 95% Confidence Interval | |
|  |  |  |  |  | Lower Bound | Upper Bound |
| 1.00 | 2.00 | .01700 | .07226 | .995 | -.1779 | .2119 |
|  | 3.00 | -.29200^*^ | .07226 | .002 | -.4869 | -.0971 |
|  | 4.00 | -.44857^*^ | .07424 | .000 | -.6488 | -.2483 |
| 2.00 | 1.00 | -.01700 | .07226 | .995 | -.2119 | .1779 |
|  | 3.00 | -.30900^*^ | .07226 | .001 | -.5039 | -.1141 |
|  | 4.00 | -.46557^*^ | .07424 | .000 | -.6658 | -.2653 |
| 3.00 | 1.00 | .29200^*^ | .07226 | .002 | .0971 | .4869 |
|  | 2.00 | .30900^*^ | .07226 | .001 | .1141 | .5039 |
|  | 4.00 | -.15657 | .07424 | .170 | -.3568 | .0437 |
| 4.00 | 1.00 | .44857^*^ | .07424 | .000 | .2483 | .6488 |
|  | 2.00 | .46557^*^ | .07424 | .000 | .2653 | .6658 |
|  | 3.00 | .15657 | .07424 | .170 | -.0437 | .3568 |
| *. The mean difference is significant at the 0.05 level.  **Table S14.** Serum BUN levels | | | | | | |
|  | | | | | | |
|  | | | | | | |
|  | | | | | | |
|  | | | | | | |

| control | 1 | 1.4 |
| --- | --- | --- |
|  | 2 | 1.366667 |
|  | 3 | 1.4 |
|  | 4 | 1.366667 |
|  | 5 | 1.2 |
|  | 6 | 1.1 |
|  | 7 | 1.033333 |
|  | 8 | 1.5 |
|  | 9 | 1.2 |
|  | 10 | 1.7 |
|  | average | 1.326667 |
|  | sd | 0.198015 |
| 10 ug/mL |  |  |
|  | 1 | 1.517073 |
|  | 2 | 1.487805 |
|  | 3 | 1.829268 |
|  | 4 | 1.9 |
|  | 5 | 1.02439 |
|  | 6 | 1.319512 |
|  | 7 | 1.04878 |
|  | 8 | 2 |
|  | 9 | 1.6 |
|  | 10 | 1.7 |
|  | average | 1.542683 |
|  | sd | 0.335496 |
|  |  |  |
| 100 ug/mL | 1 | 1.452941 |
|  | 2 | 1.7 |
|  | 3 | 1.980392 |
|  | 4 | 1.511628 |
|  | 5 | 1.767442 |
|  | 6 | 1.3 |
|  | 7 | 1.19375 |
|  | 8 | 1.8 |
|  | 9 | 1.9 |
|  | 10 | 1.7 |
|  | average | 1.630615 |
|  | sd | 0.257733 |
|  |  |  |
| 1000 ug/mL | 1 | 2.1 |
|  | 2 | 2.5 |
|  | 3 | 2.7 |
|  | 4 | 2.3 |
|  | 5 | 2 |
|  | 6 | 2 |
|  | 7 | 2.3 |
|  | 8 | 2.4 |
|  | 9 | 2.2 |
|  | average | 2.277778 |
|  | sd | 0.233333 |

| **Table S15.** Multiple Comparisons, Tukey HSD of Serum BUN | | | | | | |
| --- | --- | --- | --- | --- | --- | --- |
| Dependent Variable: VAR00002  Tukey HSD | | | | | | |
| (I) VAR00001 | (J) VAR00001 | Mean Difference (I-J) | Std. Error | Sig. | 95% Confidence Interval | |
|  |  |  |  |  | Lower Bound | Upper Bound |
| 1.00 | 2.00 | -.21602 | .11709 | .270 | -.5318 | .0998 |
|  | 3.00 | -.30395 | .11709 | .063 | -.6197 | .0118 |
|  | 4.00 | -.95111^*^ | .12030 | .000 | -1.2755 | -.6267 |
| 2.00 | 1.00 | .21602 | .11709 | .270 | -.0998 | .5318 |
|  | 3.00 | -.08793 | .11709 | .876 | -.4037 | .2279 |
|  | 4.00 | -.73509^*^ | .12030 | .000 | -1.0595 | -.4107 |
| 3.00 | 1.00 | .30395 | .11709 | .063 | -.0118 | .6197 |
|  | 2.00 | .08793 | .11709 | .876 | -.2279 | .4037 |
|  | 4.00 | -.64716^*^ | .12030 | .000 | -.9716 | -.3227 |
| 4.00 | 1.00 | .95111^*^ | .12030 | .000 | .6267 | 1.2755 |
|  | 2.00 | .73509^*^ | .12030 | .000 | .4107 | 1.0595 |
|  | 3.00 | .64716^*^ | .12030 | .000 | .3227 | .9716 |
| *. The mean difference is significant at the 0.05 level. | | | | | | |

**Acknowledgments**

J.F.T and S.M.T would like to thank the University of Windsor for providing funding for this project. This work was made possible by the facilities of the Shared Hierarchical Academic Research Computing Network (SHARCNET: www.sharcnet.ca) and Compute/Calcul Canada. M.A. and F.S. would like to thank the University of Sistan and Baluchestan for financial support for this work. A.R., M.R.H. and M.J. would like to thank the University of Zabol for financial support for this work. All authors declare that they have no competing financial interest.

**References**

[1] F. Fajaroh, H. Setyawan, W. Widiyastuti, S. Winardi, Synthesis of magnetite nanoparticles by surfactant-free electrochemical method in an aqueous system, Adv. Powder Technol., 23 (2012) 328-333.

[2] A.D. Becke, A new mixing of Hartree–Fock and local density‐functional theories, J. Chem. Phys., 98 (1993) 1372-1377.

[3] X. Ma, Z. Zhang, A. Wang, The transition of fly ash-based geopolymer gels into ordered structures and the effect on the compressive strength, Construct. Build. Mater., 104 (2016) 25-33.

[4] K. Momma, F. Izumi, VESTA 3 for three-dimensional visualization of crystal, volumetric and morphology data, J. Appl. Crystallogr., 44 (2011) 1272-1276.

[5] Performance Standards for Antimicrobial Susceptibility Testing; Seventeenth Informational Supplement., Clinical and Laboratory Standards Institute, Wayne, PA, 2007.

[6] P.R. Murray, K.S. Rosenthal, M.A. Pfaller, Medical Microbiology, Mosby/Elsevier, Philadelphia, 2009.

[7] S.-M. Hosseini-Zijoud, S.A. Ebadi, M.T. Goodarzi, M. Hedayati, R. Abbasalipourkabir, M.P. Mahjoob, J. Poorolajal, F. Zicker, N. Sheikh, Lipid Peroxidation and Antioxidant Status in Patients with Medullary Thyroid Carcinoma: A Case-Control Study, J. Clin. Diagn. Res., 10 (2016) BC04-BC07.

[8] H. Ohkawa, N. Ohishi, K. Yagi, Assay for lipid peroxides in animal tissues by thiobarbituric acid reaction, Anal. Biochem., 95 (1979) 351-358.

[9] A. Gupta, M.L.B. Bhatt, M.K. Misra, Lipid peroxidation and antioxidant status in head and neck squamous cell carcinoma patients, Oxid. Med. Cell. Longevity, 2 (2009) 68-72.
